# *Pneumocystis* pneumonia in French intensive care units in 2013–2019: mortality and immunocompromised conditions

**DOI:** 10.1186/s13613-024-01309-y

**Published:** 2024-05-22

**Authors:** Toufik Kamel, Thierry Boulain

**Affiliations:** Service de Médecine Intensive Réanimation, Centre Hospitalier Universitaire d’Orléans, 14 Avenue de L’Hôpital CS 86709, 45067 Orleans Cedex 2, France

**Keywords:** Pneumocystis pneumonia, Epidemiology, Intensive care unit, Immunodeficiency

## Abstract

**Purpose:**

The recent epidemiology of *Pneumocystis* pneumonia (PCP) requiring intensive care unit (ICU) admission and the associated spectrum of immunocompromising conditions are poorly described.

**Methods:**

We analyzed all adult PCP cases admitted to French ICUs via the French medical database system (PMSI), over the period from 2013 to 2019.

**Results:**

French ICUs admitted a total of 4055 adult patients with PCP. Among all hospitalized PCP cases, the proportion requiring ICU admission increased from 17.8 in 2014 to 21.3% in 2019 (P < 0.001). The incidence of severe PCP rose from 0.85 in 2013 to 1.32/100,000 adult inhabitants in 2019 (P < 0.0001), primarily due to the proportion of HIV-negative patients that increased from 60.6% to 74.4% (P < 0.0001). Meanwhile, the annual number of severe PCP cases among patients with HIV infection remained stable over the years. In-hospital mortality of severe PCP cases was 28.5% in patients with HIV infection and 49.7% in patients without.

Multivariable logistic analysis showed that patients with HIV infection had a lower adjusted risk of death than patients without HIV infection (Odds Ratio [OR]: 0.30, 95% confidence interval [95CI]: 0.17–0.55). Comorbidities or conditions strongly associated with hospital mortality included the patient’s age, Simplified Acute Physiologic Score II, congestive heart failure, coagulopathy, solid organ cancer, and cirrhosis. A vast array of autoimmune inflammatory diseases affected 19.9% of HIV-negative patients.

**Conclusions:**

The number of PCP cases requiring ICU admission in France has risen sharply. While the yearly count of severe PCP cases in HIV-infected patients has remained steady, this rise predominantly affects cancer patients, with a recent surge observed in patients with autoimmune inflammatory diseases, affecting one in five individuals.

**Supplementary Information:**

The online version contains supplementary material available at 10.1186/s13613-024-01309-y.

## Background

*Pneumocystis jirovecii* pneumonia (PCP) is an opportunistic fungal pneumonia that affects immunocompromised individuals [1]. Initially, it was identified in pre-term newborns in the 1950s and later in adults in the 1970s [2–6]. An epidemic phase was observed in the 1980s among patients with human immunodeficiency virus (HIV) infection [6]. This wave was greatly reduced by the introduction of antibiotic prophylaxis in the late 1980’s [6, 7] and by the advent of highly active antiretroviral therapy in the mid-1990’s [6, 8]. In the US, the prevalence of PCP among hospitalized HIV-positive patients showed a constant decline from 2002 to 2014 [9]. Despite this, PCP remains the second most common infection that defines AIDS in HIV-positive patients [10, 11]. The increasing use of immunosuppressive therapies (including corticosteroids) to treat conditions like cancer, hematologic malignancies, organ transplantation and autoimmune inflammatory diseases has led to an increased PCP incidence, with cases among patients with cancer or autoimmune diseases exceeding cases in HIV-positive patients [12–14]. While PCP is still considered a rare disease based on the European definition of fewer than five cases per 10,000 inhabitants annually, its annual number of cases worldwide could approach 500,000 cases [15]. It also remains the most common opportunistic respiratory infection in immunocompromised individuals [16], accounting for 12% of cases of community pneumonia in renal transplant patients, for example [17]. Numerous studies have shown that inflammatory diseases place patients at risk of PCP, and recommendations concerning prophylaxis have been developed in the last decades. However, comprehensive epidemiological studies that identified all potentially at-risk diseases are rare, and some inflammatory diseases still lack prophylaxis recommendations [18]. Even in the presence of prophylaxis recommendations, their application is imperfect [18, 19].

In this context, we aimed to evaluate the burden of severe PCP, defined as PCP cases requiring intensive care unit (ICU) admission during their hospitalization, in the French population during the years 2013 to 2019 by taking a comprehensive inventory of potentially predisposing diseases, comparing hospital outcome between HIV and non-HIV infected patients, and identifying the most important predisposing factors for in-hospital mortality.

## Methods

This retrospective cohort study utilized de-identified data from the national *Programme de médicalisation des systèmes d’information* (PMSI) database, which is maintained by the French national agency for the management of hospitalization data (*Agence technique de l’information sur l’hospitalisation*, ATIH). The database includes all discharge summaries of patients from both private and public acute care hospitals in France, providing information such as patient demographics, length of stay, discharge conditions (including vital status at hospital discharge), cause of admission, and underlying diseases coded according to the tenth revision of the International Classification of Diseases (ICD-10).

Our study focused on discharge summaries from the years 2013 to 2019 that contained the diagnosis of PCP (i.e., the ICD-10 codes B485, B59, or B206) and an ICU stay in patients aged 18 years or older. Along with all acute and chronic patient’s conditions, these discharge summaries also include the diagnosis declared as the main cause of ICU admission, as well as the Simplified Acute Physiology Score II (SAPS II) [20] at ICU admission. We converted the ICD-10 codes for associated diagnoses into Elixhauser classes [21], using algorithms provided by the Agency for Healthcare Research and Quality (https://hcup-us.ahrq.gov/toolssoftware/comorbidityicd10/comorbidity_icd10.jsp). We also added detailed diagnoses of autoimmune, inflammatory, rheumatoid diseases, solid organ or hematologic malignancies, and organ transplantation (ICD-10 codes used are provided in the Online Supplemental material). For patients with multiple ICU stays during hospital stay, only the last ICU admission was considered.

Furthermore, to determine the frequency of ICU admissions among all patients diagnosed with PCP, and potentially identify patient subgroups more prone to ICU admission, we gathered hospitalization data of all PCP patients, regardless of ICU utilization. However, because of privacy policy restrictions inherent to the ATIH platform at the time of data extraction, these data were not available for the year 2013.

This study adhered to the French legal regulations for observational retrospective studies involving de-identified data, which are classified as non-human subject research. The investigators were authorized by the *Commission Nationale Informatique et Liberté* (CNIL, the National Data Protection Commission) to access the national PMSI database for scientific research purposes, to extract and analyze datasets without the need to inform the patients (which would not have been possible because of the de-identification of the data). The study protocol was approved by the ethics committee of the French Society of Critical Care (#CE SRLF 23-031).

This article adheres to the STROBE guidelines for the reporting of observational studies using routinely collected health data (RECORD statement) [22].

### Statistical analysis

We expressed categorical variables as counts and percentages and continuous variables as mean (SD) or median (interquartile range) depending on their normal or non-normal distribution as graphically checked on quantile–quantile and density plots. Groups were compared by the χ^2^ test, t-test, Mann–Whitney U test, Cochran-Armitage test or analysis of variance, as appropriate. The incidence of severe adult PCP was calculated for each year using the national demographic data that are made publicly available by the National Institute of Statistics and Economic Studies (INSEE) (https://www.insee.fr/fr/statistiques/).

To identify the patients’ characteristics associated with in-hospital mortality, we used a multivariable binary logistic regression model. For the selection of variables, we used augmented backward elimination (ABE) that combines the standardized change-in-estimate criterion with significance-based backward elimination [23, 24]. The procedure was parameterized to minimize the risk of eliminating important variables (see Online Supplemental material for details). All interactions of each Elixhauser category of diseases with age and with HIV infection were considered and kept in the final model if significantly associated with in-hospital death. We use the aera under the receiver operating characteristics (AUC_ROC_) curve and cross-validation to assess the discriminative power and calibration of the final logistic model, respectively.

For each variable retained in the final model, the association with in-hospital death was expressed as the adjusted odds ratio (OR) and its 95% confidence interval (95CI).

A 2-tailed *P* < 0.05 was considered statistically significant. However, P-values were not adjusted for multiple testing and should be considered exploratory. The analyses were performed using R software version 4.2.2 (R Foundation for Statistical Computing, http://www.R-project.org).

## Results

### Overall population of patients hospitalized with PCP over the period 2014–2019, and rate of ICU admission

Over the 2014–2019 period, a total of 18,306 patients were hospitalized with PCP. The number of cases increased steadily from 2821 cases in 2014 to 3119 cases in 2019, resulting in a significant rise from 5.7 to 6.2 cases per 100,000 adult inhabitants (P < 0.001) (Table 1). Concurrently, the proportion of PCP patients requiring ICU admission increased from 17.8 in 2014 to 21.3% in 2019 (P < 0.001), with 19.9% of all PCP patients (3541/18,306) admitted to the ICU over the 2014–2019 period. Notably, patients with HIV infection constituted 33.1% (6059/18,306) of PCP cases during this period, yet their proportion declined significantly each year, from 42.8 in 2014 to 27.3% in 2019 (P < 0.001). On average, patients with HIV infection were less frequently admitted to the ICU (18.1%) compared to those without HIV infection (20.7%) (P < 0.001). However, the percentage of HIV-infected patients requiring ICU admission increased significantly over the years, from 14.3 in 2014 to 19.9% in 2019 (P < 0.001). In-hospital mortality rates significantly rose from 6.5 in 2014 to 9.3% in 2019 among patients not admitted to the ICU (P < 0.001), while showing a slight and non-significant decrease among ICU-admitted patients (from 44.4 in 2014 to 40.0% in 2019; P = 0.29). (Table 1). The proportions of PCP cases admitted to the ICU did not significantly differ across various categories of immunosuppression aside from HIV infection (see Table 1).

**Table 1:** Patient numbers and clinical characteristics of hospitalized *P. jirovecii* pneumonia cases, with proportions admitted to intensive care units, 2014–2019

	Overall N = 18,306	Patients not hospitalized in ICU N = 14,665	Patients hospitalized in ICU N = 3641
*Year of hospital admission*^a^			
2014	2821	2318	503 (17.8) ^b,c^
2015	2982	2416	566 (19.0)
2016	3071	2401	670 (21.8)
2017	3150	2518	632 (20.1)
2018	3163	2557	606 (19.2)
2019	3119	2455	664 (21.3)
Age, yr	59.7 ± 14.9	59.4 ± 15.0	61.2 ± 14.4^d^
Female sex	6567	5352	1215 (18.5)^e^
Male sex	11,739	9313	2426 (20.7)
In-hospital deaths	2781 [15.2%]^f^	1211 [8.3%]^f,g^	1580 [43.4%]^f,g^
Patients with HIV infection	6059^h^	4961	1098 (18.1)^i,j^
Patients without HIV infection	12,247	9707	2540 (20.7)
Patients with hematologic malignancies (without HIV infection)	4675	3696	979 (20.9)^k^
Patients with solid organ cancer (without HIV infection)	2540	1981	559 (22.0)
Solid organ transplant recipients (without HIV infection)	1509	1208	301 (19.0)
Patients with inflammatory/autoimmune disease (without HIV infection)	1837	1406	431 (23.5)

^a^Data regarding hospitalized (any department or ward) patients with *P. jirovecii* pneumonia were not available for the years before 2014

^b^Numbers within parentheses are percentages of patients who were hospitalized in ICU among the number of hospitalized patients in each category (i.e., category specified by row name)

^c^The percentage of patients hospitalized in ICU significantly increased in parallel with the year of admission (P < 0.001)

^d^Patients hospitalized in ICU were significantly older than patients not hospitalized in ICU (P < 0.001)

^e^The percentage of patients hospitalized in ICU was significantly lower for female than for male patients (P < 0.001) over the 2014–2019 period

^f^Percentages into square brackets are percentages upon the entire population (i.e., numbers in column headings)

^g^In-hospital mortality significantly increased over the years from 6.5 in 2014 to 9.3% in 2019 in patients not hospitalized in ICU (P < 0.001), whereas it slightly and non-significantly decrease for patients hospitalized in ICU (from 44.4% in 2014 to 40.0% in 2019; P = 0.29)

^h^The percentage of patients with HIV infection significantly declined over the years, from 42.8 in 2014 to 27.3% in 2019 (P < 0.001)

^i^The percentage of patients hospitalized in ICU was significantly lower for patients with HIV infection than for patients without (P < 0.001) over the 2014–2019 period

^j^The percentage of patients hospitalized in ICU increased from 14.3 to 19.9% over the years among patients with HIV infection (P < 0.001), whereas it did not change significantly among patients without HIV infection (20.5% in 2014 and 21.8% in 2019; P = 0.27)

^k^There was no significant difference in the percentages of patients hospitalized in ICU among the different categories of diseases (P = 0.055)

### PCP cases admitted to the ICU over the period 2013–2019

**Fig. 1:**
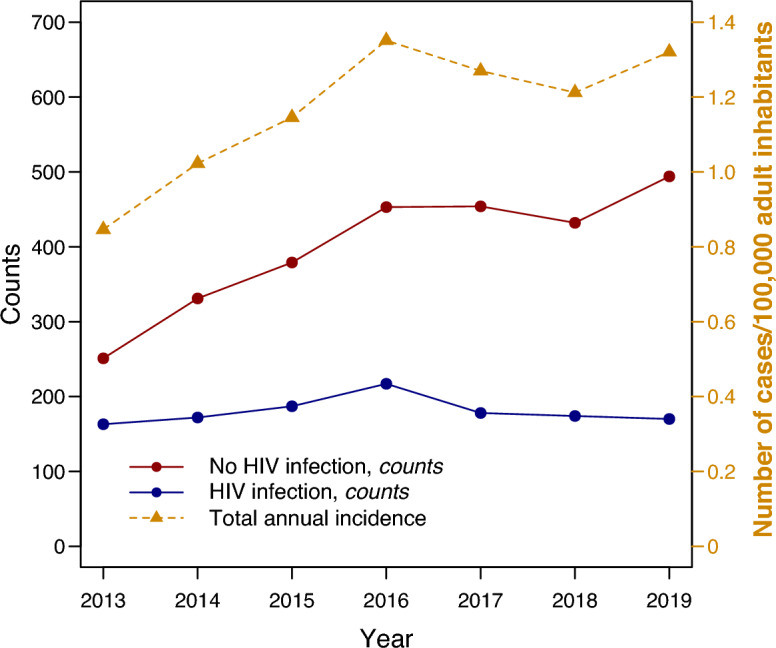
Annual incidence of Pneumocystis pneumonia admitted in an intensive care unit at the national level

Over the 2013–2019 period, 4,055 adult patients had a diagnosis of PCP and were admitted to an ICU during their hospital stay. They had a mean age of 60.8 (SD:14.6) years and 66.5% were male. There were 1,261 (31.1%) patients with HIV infection, who were significantly younger and were more frequently male than the non-HIV patients (Table 2). The incidence of severe PCP significantly increased from 0.85/100,000 in 2013 to 1.32/100,000 adult inhabitants in 2019 (P < 0.0001), a 55.3% percent rise related to the progression of cases in patients with other cause of immunosuppression than HIV infection. The proportion of patients without HIV infection significantly increased from 251/414 (60.6%) to 494/664 (74.4%) between 2013 and 2019 (P < 0.0001) (Fig. 1).

**Table 2:** Demographic and clinical characteristics of 4,055 patients with severe *P. jirovecii* pneumonia, 2013–2019

	Overall N = 4055	HIV-negative patients N = 2794	Patients with HIV infection N = 1261	P-value
*Year of hospital admission*				< 0.001
2013	414 (10.2)	251 (9.0)	163 (12.9)	
2014	503 (12.4)	331 (11.8)	172 (13.6)	
2015	566 (14.0)	379 (13.6)	187 (14.8)	
2016	670 (16.5)	453 (16.2)	217 (17.2)	
2017	632 (15.6)	454 (16.2)	178 (14.1)	
2018	606 (14.9)	432 (15.5)	174 (13.8)	
2019	664 (16.4)	494 (17.7)	170 (13.5)	
Age, yrs (mean (SD))	60.8 (14.7)	64.8 (13.4)	52.0 (13.3)	< 0.001
Female sex	1357 (33.5)	992 (35.5)	365 (28.9)	< 0.001
Hospital length of stay, days (median [IQR])	23 [14, 38]	21 [13, 36]	27 [16, 42]	< 0.001
Previous ICU admission in same hospital stay	469 (11.6)	128 (4.6)	341 (27.0)	< 0.001
SAPS II (mean [SD]) (missing: n = 3 [0.1%])	47.3 (17.2)	47.3 (17.2)	47.5 (17.2)	0.746
Length of ICU stay, days (median [IQR])	10	10 [5, 18]	9 [4, 19]	0.010
Deaths in ICU	1540 (38.0)	1225 (43.8)	315 (25.0)	< 0.001
Deaths in hospital	1748 (43.1)	1389 (49.7)	359 (28.5)	< 0.001
Organ failure or disease declared at ICU admission				
Acute respiratory failure	3219 (79.4)	2243 (80.3)	976 (77.4)	0.040
Acute respiratory distress syndrome	1674 (41.3)	1167 (41.8)	507 (40.2)	0.368
Septic shock	121 (3.0)	87 (3.1)	34 (2.7)	0.533
Other shock	29 (0.7)	20 (0.7)	9 (0.7)	> 0.99
Acute renal failure	42 (1.0)	29 (1.0)	13 (1.0)	> 0.99
Meningitis or meningo-encephalitis	15 (0.4)	2 (0.1)	13 (1.0)	< 0.001
Neutropenia	14 (0.3)	12 (0.4)	2 (0.2)	0.284
Altered consciousness	8 (0.2)	6 (0.2)	2 (0.2)	> 0.99
Acute liver failure	8 (0.2)	6 (0.2)	2 (0.2)	> 0.99

*ICU* intensive care unit, *IQR* interquartile range, *SAPS II* Simplified Acute Physiology Score II, *SD* standard deviation

Chronic cardiac diseases, chronic respiratory diseases and diabetes were significantly more prevalent in HIV-negative patients than in patients with HIV infection (Table S1).

### Hospital and intensive care unit mortality

**Fig. 2:**
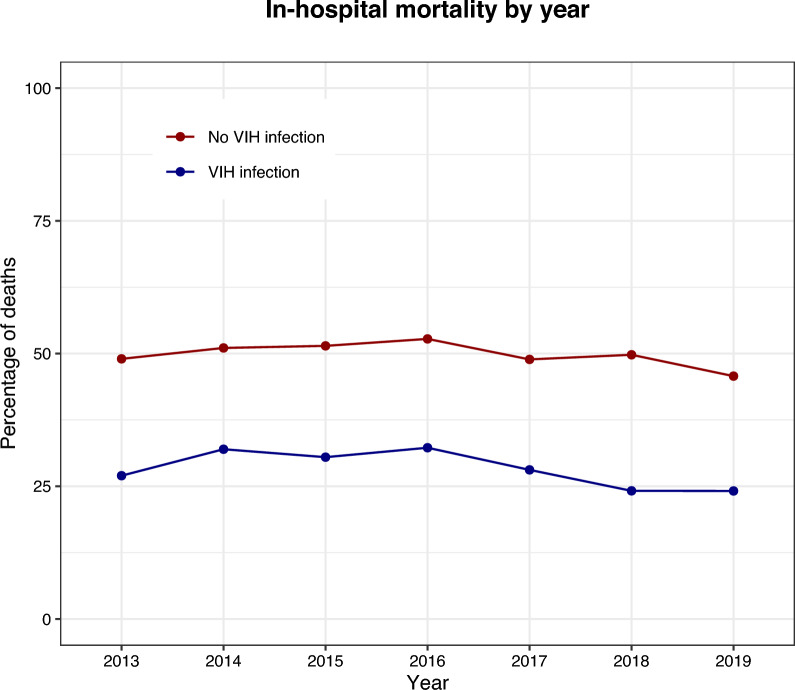
Annual death rate

The in-hospital mortality rate was 43.1% over the study period. It was significantly lower in patients with HIV infection (28.5%) than without (49.7%) (P < 0.0001) (Table 2). There was no significant change in in-hospital mortality between years, either in patients with or without HIV infection (P = 0.14 for both cohorts) (Fig. 2).

Among hospital decedents, 88.1% died in the ICU. Death rate in the ICU was 38.0% over the entire study period and did not change significantly between years (P = 0.48). Patients with HIV infection less often died in the ICU (25.0%) than patients without (43.8%) (P < 0.0001) (Table 2).

The severity of disease on admission to the ICU, as expressed by the SAPSII score, remained stable over the years (46.4 ± 16.5 in 2013 to 47.1 ± 16.4 in 2019; P = 0.44), and was not different between patients with and without HIV infection (P = 0.67).

In severe PCP cases not related to HIV infection, the hospital mortality was consistently 10 percentage points higher than the mortality predicted by the SAPSII score, while SAPSII consistently overestimated hospital mortality by 10 to 20 percentage points for patients with HIV infection (Figure S1).

### Causes of immunosuppression

**Fig. 3:**
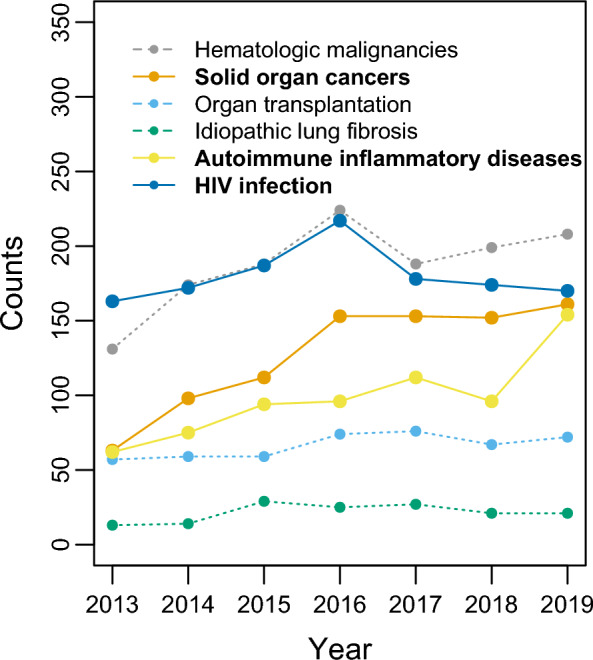
Causes of immunosuppression by year. Solid lines and text in bold letters correspond to conditions or diseases whose proportions showed significant changes over the study period (see text)

Among the 4,055 included patients, the percentage of HIV infection steadily and significantly declined from 2013 to 2019 and the percentage of solid organ cancer grew significantly (P < 0.001) to reach 24.2% in 2019. Meanwhile the proportion of autoimmune inflammatory diseases showed a significant rise only between 2018 and 2019 (P < 0.001), reaching 23.2% in 2019 (Fig. 3).

Over the study period, immunodeficiency in HIV-negative patients was caused by hematologic malignancies (38.9%), solid organ cancer (21.7%), autoimmune inflammatory diseases (19.9%), organ transplantation (12.3%), and idiopathic lung fibrosis (4.3%) (Table S2). Almost all types of hematologic malignancies were represented (Table S2).

Among the autoimmune inflammatory diseases recorded, rheumatoid arthritis, ulcerative colitis, Crohn's disease, psoriasis, and polymyalgia rheumatica affected 10.3% of patients. All other autoimmune inflammatory diseases, considered rare diseases, affected 13.2% of HIV-negative patients.

No cause of immunodeficiency was recorded for 286 patients, representing 7.1% of the overall population and 10.2% of patients without HIV infection. Among the later, chronic respiratory diseases (17.1% vs. 11.8%; P = 0.011) and cirrhosis (22.4% vs. 3.3%; P < 0.001) were significantly more prevalent than in patients with a recorded cause of immunodeficiency (Table 3).

**Table 3:** Comorbidities in patients with severe *P. jirovecii* pneumonia and identified or non-identified cause of immunodeficiency

	Immunocompromising condition identified		
	Yes	No	P-value
	N = 3769	N = 286	
Age, yr (mean [SD])	60.4 (14.6)	65.9 (14.6)	< 0.001
Female sex	1260 (33.4)	97 (33.9)	0.918
SAPS II (mean [SD])	47.2 (17.1)	49.1 (18.9)	0.081
In-hospital mortality	1570 (41.7)	178 (62.2)	< 0.001
In-ICU mortality	1370 (36.3)	170 (59.4)	< 0.001
Congestive heart failure	592 (15.7)	58 (20.3)	0.051
Cardiac arrhythmias	844 (22.4)	75 (26.2)	0.156
Valvular disease	112 (3.0)	8 (2.8)	> 0.99
Peripheral vascular disorders	117 (3.1)	8 (2.8)	0.911
Hypertension	1049 (27.8)	88 (30.8)	0.318
Pulmonary circulation disorders	62 (1.6)	14 (4.9)	< 0.001
Chronic pulmonary disease	446 (11.8)	49 (17.1)	0.011
COPD*	332 (8.8)	36 (12.6)	0.042
Chronic respiratory failure*	211 (5.6)	25 (8.7)	0.040
Liver disease	366 (9.7)	68 (23.8)	< 0.001
Cirrhosis*	124 (3.3)	64 (22.4)	< 0.001
Alcohol abuse	155 (4.1)	38 (13.3)	< 0.001
Paralysis	206 (5.5)	14 (4.9)	0.783
Other neurological disorders	257 (6.8)	17 (5.9)	0.656
Diabetes uncomplicated	483 (12.8)	44 (15.4)	0.248
Diabetes complicated	157 (4.2)	12 (4.2)	> 0.99
Hypothyroidism	158 (4.2)	16 (5.6)	0.329
Renal failure	657 (17.4)	32 (11.2)	0.009
Chronic dialysis*	109 (2.9)	6 (2.1)	0.552
Peptic ulcer	74 (2.0)	9 (3.1)	0.252
Coagulopathy	577 (15.3)	35 (12.2)	0.189
Obesity	233 (6.2)	22 (7.7)	0.375
Weight loss	1417 (37.6)	62 (21.7)	< 0.001
Fluid electrolyte disorders	1293 (34.3)	90 (31.5)	0.362
Blood loss anemia	188 (5.0)	13 (4.5)	0.848
Deficiency anemias	716 (19.0)	26 (9.1)	< 0.001

*COPD* chronic obstructive pulmonary disease, *ICU* intensive care unit, *SAPS II* Simplified Acute Physiology Score II, *SD* standard deviation

Elixhauser categories were used for classifying comorbidities. We added some sub-categories or conditions, which are marked with an asterisk (*)

Among HIV-positive patients, causes of immunodeficiency other than HIV infection were not infrequent (Table S2).

### Factors associated with in-hospital mortality

The final logistic model retained to identify predictors of in-hospital death was constructed on a population of 4052 patients instead of 4055, because 3 patients (< 0.1%) had no SAPS II value recorded, which we did not replace. The model had a discriminative power as assessed by the AUC_ROC_ of 0.80 (95CI: 0.78–0.81) and was well calibrated (Figure S2).

Patient’s age (OR: 1.15 [95CI: 1.12–1.19]; per 1-yr increase), SAPSII score (OR: 1.04 [95CI: 1.03–1.04]; per 1-point increase) on admission to the ICU, and the number of hospital wards in which the patient stayed before being admitted to the ICU (OR: 1.19 [95CI: 1.12–1.27]) were associated with an increased risk of death (Table 4).

**Table 4:** Multivariable logistic model for in-hospital mortality in 4052 patients with severe* P. jirovecii* pneumonia

	Odds ratio (95% confidence interval)	P-value
Year of hospitalization (for 1-yr after 2013)	0.94 (0.91–0.98)	0.00314
Age (per 1-yr increment)	1.15 (1.12–1.19)	< 0.00001
SAPSII (per 1-point increment)	1.04 (1.03–1.04)	< 0.00001
Congestive heart failure	23.17 (8.34–64.38)	< 0.00001
Solid organ cancer (with or w/o metastasis)	6.56 (2.55–16.86)	0.00009
HIV infection	0.30 (0.17–0.55)	0.00007
Cirrhosis	3.30 (2.20–4.95)	< 0.00001
Hematologic malignancies	0.44 (0.25–0.77)	0.00388
Sex male	1.16 (0.99–1.36)	0.06
Inflammatory and autoimmune diseases	1.23 (0.98–1.54)	0.08
Chronic pulmonary disease	0.87 (0.69–1.09)	0.22
Renal failure	0.85 (0.66–1.09)	0.20
Coagulopathy	9.98 (3.90–25.54)	< 0.00001
Esophagus or gastric cancer	2.95 (1.55–5.63)	0.00099
Idiopathic lung fibrosis	2.78 (1.86–4.16)	< 0.00001
Lung cancer	2.48 (1.75–3.52)	< 0.00001
Allogenic stem cell transplantation	1.99 (1.33–2.97)	0.00074
Dementia	1.99 (1.12–3.52)	0.01807
Lack of identified cause of immunosuppression	1.80 (1.30–2.49)	0.00042
Brain cancer	1.75 (0.90–3.39)	0.10
Septic shock on ICU admission	1.56 (1.01–2.41)	0.049
Solid organ transplantation	1.21 (0.58–2.50)	0.61
Rank of ICU admission among admissions to successive wards during same hospitalization	1.19 (1.12–1.27)	< 0.00001
Obesity	0.74 (0.55–1.00)	0.049
Kidney transplantation	0.71 (0.33–1.53)	0.38
Alcohol abuse	0.69 (0.47–1.01)	0.06
Myelodysplastic syndrome	0.65 (0.37–1.14)	0.13
Dependency	0.62 (0.46–0.84)	0.00179
Previous ICU admission during same hospitalization	0.62 (0.47–0.82)	0.00091
Psychoses	0.59 (0.46–0.76)	0.00005
Peptic ulcer	0.56 (0.34–0.94)	0.03
Multiple myeloma	0.56 (0.35–0.89)	0.01392
Weight loss	0.54 (0.46–0.63)	< 0.00001
Heart transplantation	0.53 (0.20–1.39)	0.20
Depression	0.50 (0.32–0.76)	0.00134
Acute lymphoblastic leukemia	0.45 (0.23–0.88)	0.02022
Liver transplantation	0.36 (0.15–0.84)	0.01815
Interaction « HIV infection x hematologic malignancies»		0.00021
Interaction “age x solid organ cancer”		0.00295
Interaction “age x coagulopathy»		0.00008
Interaction “age x congestive heart failure”		< 0.00001

*SAPS II* Simplified Acute Physiology Score II

In contrast with the above unadjusted analysis, the risk of in-hospital death, when adjusted for all other covariables, has decreased significantly over time (OR: 0.94 [95CI: 0.91–0.98]; for each year after 2013).

The comorbidities with the strongest association with in-hospital mortality were congestive heart failure (OR: 23.17 [95CI: 8.34–64.38]), coagulopathy (OR: 9.98 [95CI: 3.90–25.54]), solid organ neoplasm (with or without metastasis) (OR: 6.56 [95CI: 2.55–16.86]), and cirrhosis (OR: 3.30 [95CI: 2.20–4.95]).

Patients with HIV infection had a lower adjusted risk of death than patients without HIV infection (OR: 0.30 [95CI: 0.17–0.55]).

In the entire study population, hematologic malignancies were found to be associated with a reduced risk of death (OR: 0.44 [95CI: 0.25–0.77]) and to interact with the presence of HIV infection (Table 4). This association was primarily driven by the larger subset of patients without HIV infection. In contrast, patients with HIV infection who also had hematologic malignancy had a higher risk of death (see interaction plot in Figure S3).

There was a strong interaction between age and congestive heart failure (Table 4), whereby the presence of congestive heart failure placed patients at a high risk of death of roughly 40–60% irrespective of age, while in patients without congestive heart failure, the risk of death grew in parallel with age (Figure S4). Somewhat similar interactions were found between the presence of coagulopathy and age (Figure S5) and between the presence of solid organ cancer and age (Figure S6).

Septic shock was the only acute condition present at ICU admission to be retained in the logistic model as associated with in-hospital mortality (OR: 1.56 [95CI: 1.01–2.41]).

## Discussion

In France, there was an increase in the number of hospitalized cases of PCP over the study period, observed in both overall PCP cases from 2014 to 2019 and in those admitted to the ICU between 2013 and 2019. This rise was attributed to the consistent increase in the incidence of the disease among HIV-negative patients and an increase in the proportion of PCP cases in patients with HIV infection necessitating ICU admission.

In patients admitted to the ICU, the in-hospital mortality rate was high (43.1%) and remained stable throughout the study period. HIV-infected patients had a lower mortality rate compared to non-HIV-infected patients.

In patients admitted to the ICU, the risk of in-hospital death was associated with the patients’ age and disease severity at ICU admission, but also with coexisting comorbidities, such as congestive heart failure, solid organ cancer, and cirrhosis. This study identified a vast array of autoimmune inflammatory diseases as the cause of immunodeficiency.

Our finding of an overall increased incidence of PCP cases aligns with other recent epidemiological studies conducted at the national level in England, Germany, Spain, and Norway [25–28].

We noted a significant increase in the proportion of patients hospitalized in the ICU in parallel with the year of admission, rising from 17.8 in 2014 to 21.3% in 2019. These percentages are lower than in a French study conducted in the 1990’s where half of the hospitalized patients with PCP required ICU admission [29], or the ICU admission rate of 36.6% reported in another French study conducted from 2007 to 2010 [30]. More recently, a German study reported an ICU admission rate of 44% [26]. Discrepancies in these rates may be attributed to changes in the general care approach to PCP cases over the years in France, as well as differing admission policies across countries. In any case, the increase in ICU admissions of patients with PCP still imposes a significant burden on the French healthcare system.

A high mortality rate was observed among adult patients with severe PCP, which was found to be higher in HIV-negative patients (49.7%) compared to those with HIV infection (28.5%). This finding was consistent with some small, retrospective studies that focused on PCP in the ICU. For instance, one study conducted in France during the late 1990s and early 2000s reported an ICU mortality rate of 29% for all PCPs, with higher rates observed in HIV-negative patients (48%) than in patients with HIV infection (17%) [31]. Another study conducted in the US during the same period reported in-hospital mortality of 67% for 30 HIV-negative patients [32]. In 2013, a study combining three small retrospective cohorts from the same period found an in-hospital mortality rate of 25% for HIV-negative patients with PCP [33]. In the largest, more recent retrospective study that included 554 patients with PCP, regardless of ICU admission, the in-hospital mortality was 4% in patient with HIV infection and 27% without [30]. Currently, there is no available recent data on hospital mortality rates for patients with PCP admitted to the ICU.

Our findings confirm that severe PCP nowadays affects more HIV-negative patients than patients with HIV infection, particularly cancer patients [12–14, 34]. HIV-negative patients had a higher in-hospital mortality rate compared to HIV-positive patients with PCP. This observation is consistent with other studies that have examined hospital outcomes of all PCP patients admitted, regardless of ICU status [1, 30, 31, 34, 35]. Notwithstanding the fact that immunodeficiency in HIV-negative patients was primarily due to hematologic or solid organ malignancies, worse outcome in these patients may also be ascribed to severe comorbidities like chronic cardiac and respiratory diseases that were more prevalent than in patients with HIV infection (see Table S1). It is important to note that the predominant proportion of HIV-negative patients among severe PCP cases should not obscure the fact that the number of severe PCP cases in patients with HIV infection remains stable (Fig. 1), highlighting the ongoing HIV epidemic as a continuing major public health concern.

The most common immunocompromising condition observed in our cohort of severe PCP patients was hematologic malignancies, which is consistent with findings in hospitalized patients with PCP in general [36]. It is worth noting that almost all types of hematologic malignancies were present, including acute and chronic myeloid leukemias, which accounted for 44% of hematologic malignancies in HIV-negative patients (Table S2). As also observed in many studies [1, 25–27], solid organ cancer was the second most prevalent underlying condition in our population.

Autoimmune inflammatory diseases accounted for approximately 20% of the underlying conditions associated with severe PCP in HIV-negative patients. This proportion has exhibited a steady rise in several studies conducted over the last two decades [12, 28, 30, 34, 35]. This group of conditions encompasses a diverse set of rare diseases that often require immunosuppressive or immunomodulatory therapy. However, recommendations for PCP prophylaxis have been scarce, were only recently established [37–39], and are subject to debate [40], in contrast to the well-established guidelines for hematologic or solid organ malignancies and transplant recipients [41–47]. These findings collectively suggest a need for more systematic consideration of PCP antibiotic prophylaxis in these patient populations.

For 4.3% of HIV-negative patients, idiopathic lung fibrosis, which is a rare disease [48], was the sole condition that may make them more susceptible to PCP. Although it does not strictly weaken the immune system, it frequently necessitates the use of immunosuppressive treatment, such as high-dose corticosteroids. Some researchers also have recognized idiopathic lung fibrosis as an independent risk factor for PCP [35, 49–51] and as a risk factor for death from PCP [52] as observed in the present study (Table 4).

Our cohort of severe PCP cases comprised 7.1% patients with no identified cause of immunodeficiency. This proportion was less than that observed in recent Spanish (14.5%) and German (16.8%) nationwide studies also based on coding data [26, 27]. Although this proportion may still have been due to imperfect coding, it is noteworthy that patients with no identified cause of immunodeficiency suffered more frequently from chronic respiratory diseases or cirrhosis. These two conditions might be added to the list of conditions predisposing to PCP, as already suggested by others [53, 54].

This study has some limitations. First, the data relied on ICD-10 codes and administrative records, which may have resulted in inaccuracies when estimating the incidence and proportions of specific diseases. While this limitation typically has minimal impact on the outcomes of population-based studies [55], we cannot dismiss the possibility of such inaccuracies. For instance, as shown in Table S2, we observed 65 cases of PCP in patients with solid organ cancer and HIV infection, whereas such occurrences were considerably less frequent in a prospective cohort analysis conducted across 23 French hospitals over a 14-year period [56]. Second, the study did not provide details about the ICU procedures employed, such as mechanical ventilation, renal replacement therapy, or vasopressor use. However, this did not affect the estimation of in-hospital mortality. Third, the data did not allow for the calculation of the proportion of patients who received antibiotic prophylaxis for PCP, precluding an estimation of how the prophylaxis guidelines were followed. Fourth, the study, like other nationwide studies based on administrative records, did not have information about the type of sampling and laboratory test used for the diagnosis of PCP. Since the 2000s, PCR testing on respiratory samples has become increasingly utilized for diagnosing PCP [28]. However, while PCR is highly sensitive in detecting *P. jirovecii*, its specificity in confirming PCP rather than colonization in patients without HIV infection is suboptimal [57]. Large-scale prospective data are lacking to determine the proportion of true, confirmed PCP cases among all instances reported as such, both in ICUs and in the general hospitalized patient population. Hence, our finding that the incidence of PCP is increasing in non-HIV patients, while aligning with most recent epidemiological studies conducted at the national level in developed countries [25–28], warrants careful consideration until large-scale prospective studies are undertaken. Fifth, despite being recent, our data did not extend to the years 2020, 2021 and 2022. This was a deliberate decision because in many French hospitals, units administering intensive care were variable in size, location, and target population during the COVID-19 pandemic. Such variability had the potential to distort administrative data and affect ICU admission policies for patients with respiratory issues unrelated to COVID-19.

## Conclusions

There is a rise in the number of severe PCP cases requiring ICU admission in France. The mortality rate is high for these patients, especially those without HIV-related PCP. One in five patients had an underlying autoimmune inflammatory disease, suggesting a need for systematic consideration of PCP antibiotic prophylaxis in these patients.

### Supplementary Information

Additional file 1.

## Data Availability

The authors are not authorized by French law to export data extracted and analyzed within the secure ATIH platform to other sites. ATIH is the only party that can authorize data sharing with users they have not already authorized. All requests for data access must therefore be addressed directly to ATIH (https://www.atih.sante.fr/nous-contacter).

## Abbreviations

ABE: Augmented backward elimination
ATIH: *Agence technique de l’information sur l’hospitalisation*
CNIL: *Commission Nationale Informatique et Liberté*
COPD: Chronic obstructive pulmonary disease
HIV: Human immunodeficiency virus
ICD-10: Tenth revision of the International Classification of Diseases
ICU: Intensive care unit
IQR: Interquartile range
PCP: *Pneumocystis jirovecii* Pneumonia
PMSI: *Programme de médicalisation des systèmes d’information*
SAPS II: Simplified Acute Physiology Score II
