# Supplementary material for: Pneumocystis pneumonia in French intensive care units in 2013–2019: mortality and immunocompromised conditions
Source: Ann Intensive Care. 2024 May 22;14:80. doi: 10.1186/s13613-024-01309-y (PMC11111439; doi:10.1186/s13613-024-01309-y)
Supplement: Supplementary file 1 — Additional file 1. [file 13613_2024_1309_MOESM1_ESM.docx]

***Pneumocystis* pneumonia in French intensive care units in 2013-2019: Mortality and immunocompromised conditions.**

**ADDITIONAL FILE 1**

- ICD-10 codes used to identify autoimmune, inflammatory, rheumatoid diseases, solid organ or hematologic malignancies, and organ transplantation, page 2
- Details of method used for variables selection, page 4
- Table S1: Comparison of patients’ characteristics according to HIV status in 4,055 patients with severe *P. jirovecii* pneumonia, page 5
- Figure S1: Observed versus predicted in-hospital mortality, page 7
- Table S2: Inventory of immunocompromising conditions in 4,055 patients with severe *P. jirovecii* pneumonia, page 8-9
- Figure S2: Cross-validation of the multivariable, binary logistic model, page 10
- Figure S3, S4 & S5: Interactions, page 11-12
- References, page 13

**ICD-10 codes used to identify autoimmune, inflammatory, rheumatoid diseases, solid organ or hematologic malignancies, and organ transplantation**

**Autoimmune, inflammatory, rheumatoid diseases**

| Ankylosing spondylarthritis | M081, M45, M46 |
| --- | --- |
| Autoimmune hepatitis | K754 |
| Behcet disease | M352 |
| Crohn disease | K50 |
| Cryoglobulinemia | D891 |
| Fibromyalgia | M797 |
| Horton disease | M315, M316 |
| Idiopathic thrombocytopenic purpura | D693 |
| Mixed connective tissue disease | M351, M358, M359 |
| Myasthenia gravis | G700 |
| Pemphigus vulgaris | L100 |
| Polymyalgia rheumatica | M353 |
| Polymyositis or dermato-polymyositis | M331, M332, M339 |
| Psoriasis | L400, L401, L402, L403, L404, L408, L409 |
| Psoriatic arthritis | M07 |
| Rheumatoid arthritis | M05, M06, M08 |
| Sarcoidosis | D86, D860, D861, D862, D868, D869 |
| Sjogren disease | M350 |
| Still disease | M061 |
| Systemic lupus erythematosus | L930, L931, M32 |
| Systemic scleroderma | M342, M341, M340, M349, M348, M34 |
| Ulcerative colitis | K51 |
| Wegener disease and other necrotizing angiitis | M30, M31 |
| Nephrotic/nephritic syndrome | If none of the above codes and at least one of the following:  N00, N000, N001, N002, N003, N004, N005, N006, N007, N008, N009, N01, N010, N011, N012, N013, N014, N015, N016, N017, N0170, N0179, N018, N019, N03, N030, N031, N0310, N0319, N032, N033, N0330, N0339, N034, N035, N036, N037, N038, N0380, N0389, N039, N05, N050, N051, N052, N053, N054, N055, N056, N057, N058, N059,  N040, N041, N042, N043, N044, N045, N046, N047, N048, N049 |

**Solid organ transplantation**

| Kidney transplantation | Z940, T861 |
| --- | --- |
| Liver transplantation | Z944 |
| Heart transplantation | Z941, T862 |
| Lung transplantation | Z942 |
| Other transplantation | Z9481, Z9488, Z949 |

**Hematologic malignancies**

| *Lymphoma* |  |
| --- | --- |
| Non follicular lymphoma | C83 |
| Diffuse large B cell lymphoma | C833 |
| Hodgkin disease | C81 |
| Mature T NK cell lymphoma | C84 |
| Follicular lymphoma | C82 |
| Burkitt lymphoma | C837 |
| Other lymphomas | C85, C86 |
| *Leukemia* |  |
| Chronic myeloid leukemia | C921, C922 |
| Chronic lymphoid leukemia | C911 |
| Acute myeloblastic leukemia | C920 |
| Acute lymphoblastic leukemia | C910 |
| Other myeloid leukemia | C923, 924, C925, 926, C927, 928, C929 |
| Other lymphoid leukemia | C912, C913, C914, C915, C916, C917, C918, C919 |
| Chronic myelomonocytic leukemia | C931 |
| Acute mono blastic/cytic leukemia | C930 |
| Other monocytic leukemia | C933, C937, C939 |
| Other leukemia | C94, C95 |
| Histiocytosis | C96 |
| Multiple myeloma | C90 |
| Myelodysplastic syndrome | D46 |
| Myelofibrosis | D474 |
| Essential thrombocythemia | D473 |
| Other malignant immunoproliferative diseases | C88 |
| Kaposi’s sarcoma | C46 |
| Autologous stem cell transplantation | Z94800 |
| Allogenic stem cell transplantation | Z94801, Z94802, Z94803, Z94804, Z94809, Z94800 |

**Solid organ cancers**

| Lung cancer | C34 |
| --- | --- |
| Breast cancer | C50 |
| Eso gastric cancer | C15, C16 |
| Prostatis cancer | C61 |
| Anorectal, colon cancer | C18, C19, C20, C21 |
| Liver gallbladder, biliary duct, pancreas cancer | C22, C23, C24, C25 |
| Brain cancer | C71 |
| Kidney cancer | C64, C66 |
| Skin melanoma | C43 |
| Bladder cancer | C67 |
| Larynx cancer | C32 |
| Oropharynx cancer | C10, C11, C12, C14 |
| Small bowel cancer | C17 |
| Ovary cancer | C56 |
| Corpus uteri cancer | C54, C55 |
| Cervix uteri cancer | C53, C539, B977 |

**Details of method used for variables selection**

To identify the patients’ characteristics associated with in-hospital mortality, we used a multivariable binary logistic regression model. For the selection of variables, we used augmented backward elimination (ABE) that combines the standardized change-in-estimate criterion with significance-based backward elimination [1,2]. The procedure was parameterized to minimize the risk of eliminating important variables: We increased the thresholds for including variables based on significance (P-value of <0.35 to be kept in the model) and we tolerated a change-in-estimate of < 35%, as to minimize the risk of eliminating important explanatory variables. We chose to select in the first time 11 “passive” variables that we thought, based on background knowledge, they are linked to in-hospital death (namely, patient age, sex, SAPSII score, HIV infection, hematologic malignancy, solid organ cancer [with or without metastasis], inflammatory and autoimmune diseases, congestive heart failure, chronic pulmonary disease, chronic renal failure and cirrhosis), and to always leave them in the model [.]. Additionally, we forced the variable “Year of hospitalization” to assess the evolution of in-hospital mortality between 2013 and 2019. All other conditions or diseases, considered as “active” variables that could be linked to in-hospital death or could modify the influence of the passive variables, were also introduced in the global, starting model submitted to ABE. This procedure was repeated on 1000 bootstrap samples (with replacement) of the study population. We chose a priori to retain as potentially explanatory variables those that were selected in more than half of the 1000 bootstrap samples, that showed a root mean square difference (RMSD, root mean square difference between the bootstrapped regression coefficients and the regression coefficients of the global model) ratio < 1.5 and whose inclusion in the model modified the coefficients of the passive variables by less than 50% compared to the global starting model (i.e., an absolute relative conditional bias [1] of less than 50%).

**Table S1 Patients comorbidities according to HIV status in 4,055 patients with severe *P. jirovecii* pneumonia**

|  | **HIV-negative patients**  **N=2794** | **Patients with HIV infection**  **N=1261** | P-value |
| --- | --- | --- | --- |
| Congestive heart failure | 517 (18.5) | 133 (10.5) | <0.001 |
| Cardiac arrhythmias | 741 (26.5) | 178 (14.1) | <0.001 |
| Valvular disease | 95 (3.4) | 25 (2.0) | 0.018 |
| Hypertension | 911 (32.6) | 226 (17.9) | <0.001 |
| Peripheral vascular disorders | 103 (3.7) | 22 (1.7) | 0.001 |
| Ischemic cardiomyopathy | 216 (7.7) | 48 (3.8) | <0.001 |
| Atrial fibrillation | 479 (17.1) | 87 (6.9) | <0.001 |
| Chronic pulmonary disease | 368 (13.2) | 127 (10.1) | 0.006 |
| Pulmonary circulation disorders | 61 (2.2) | 15 (1.2) | 0.042 |
| COPD | 275 (9.8) | 93 (7.4) | 0.013 |
| Idiopathic lung fibrosis | 121 (4.3) | 29 (2.3) | 0.002 |
| Chronic respiratory failure | 194 (6.9) | 42 (3.3) | <0.001 |
| Home ventilation | 21 (0.8) | 3 (0.2) | 0.080 |
| Tobacco use | 185 (6.6) | 154 (12.2) | <0.001 |
| Paralysis | 152 (5.4) | 68 (5.4) | >0.99 |
| Other neurological disorders | 161 (5.8) | 113 (9.0) | <0.001 |
| Stroke sequella | 31 (1.1) | 11 (0.9) | 0.601 |
| Dementia | 32 (1.1) | 35 (2.8) | <0.001 |
| Diabetes uncomplicated | 406 (14.5) | 121 (9.6) | <0.001 |
| Diabetes complicated | 125 (4.5) | 44 (3.5) | 0.172 |
| Hypothyroidism | 143 (5.1) | 31 (2.5) | <0.001 |
| Renal failure | 523 (18.7) | 166 (13.2) | <0.001 |
| Chronic dialysis | 83 (3.0) | 32 (2.5) | 0.505 |
| Liver disease | 260 (9.3) | 174 (13.8) | <0.001 |
| Cirrhosis | 145 (5.2) | 43 (3.4) | 0.016 |
| Alcohol abuse | 118 (4.2) | 75 (5.9) | 0.021 |
| Peptic ulcer | 57 (2.0) | 26 (2.1) | >0.99 |

**Table S1 (continued)**

|  | **HIV-negative patients**  **N=2794** | **Patients with HIV infection**  **N=1261** | P-value |
| --- | --- | --- | --- |
| Solid organ transplantation | 345 (12.3) | 119 (9.4) | 0.008 |
| Hematologic malignancies | 1087 (38.9) | 223 (17.7) | <0.001 |
| Solid organ cancer | 606 (21.7) | 65 (5.2) | <0.001 |
| Including Metastatic cancer | 362 (13.0) | 33 (2.6) | <0.001 |
| Autoimmune inflammatory diseases | 555 (19.9) | 134 (10.6) | <0.001 |
| Coagulopathy | 437 (15.6) | 175 (13.9) | 0.160 |
| Obesity | 220 (7.9) | 35 (2.8) | <0.001 |
| Weight loss | 871 (31.2) | 608 (48.2) | <0.001 |
| Fluid electrolyte disorders | 968 (34.6) | 415 (32.9) | 0.297 |
| Blood loss anemia | 128 (4.6) | 73 (5.8) | 0.118 |
| Deficiency anemias | 509 (18.2) | 233 (18.5) | 0.877 |
| Drug abuse | 10 (0.4) | 47 (3.7) | <0.001 |
| Psychoses | 278 (9.9) | 188 (14.9) | <0.001 |
| Depression | 106 (3.8) | 55 (4.4) | 0.441 |

ICU, Intensive care unit; IQR, interquartile range; SAPS II, Simplified Acute Physiology Score II; SD, standard deviation

**Figure S1**

**A B**

**Figure S1 caption:**

**Title:** Observed versus predicted mortality in patients with and without HIV infection

**Legend**:

Panel A, patients without HIV infection; Panel B, patients with HIV infection

SAPS II, Simplified Acute Physiology Score II.

**Table S2: Inventory of immunocompromising conditions in 4,055 patients with severe *P. jirovecii* pneumonia**

|  | **HIV-negative patients**  N=2794 | **HIV-positive patients**  N=1261 |
| --- | --- | --- |
| **HIV infection** | _ | **1261 (100.0)** |
| **Hematologic malignancies** | **1087 (38.9 ) ^a^** | **193 (15.3)** |
| *Lymphoma* |  |  |
| Non follicular lymphoma | 186 (6.7) | 34 (2.7) |
| Diffuse large B cell lymphoma | 138 (4.9) | 24 (1.9) |
| Hodgkin disease | 67 (2.4) | 17 (1.3) |
| Mature T NK cell lymphoma | 40 (1.4) | 8 (0.6) |
| Follicular lymphoma | 35 (1.3) | 6 (0.5) |
| Burkitt lymphoma | 6 (0.2) | 4 (0.3) |
| Other lymphomas | 129 (4.6) | 28 (2.2) |
| *Leukemia* |  |  |
| Chronic myeloid leukemia | 192 (6.9) | 54 (4.3) |
| Chronic lymphoid leukemia | 127 (4.5) | 9 (0.7) |
| Acute myeloblastic leukemia | 110 (3.9) | 6 (0.5) |
| Acute lymphoblastic leukemia | 58 (2.1) | 7 (0.6) |
| Other myeloid leukemia | 193 (6.9) | 25 (2.0) |
| Other lymphoid leukemia | 26 (0.9) | 6 (0.5) |
| Chronic myelomonocytic leukemia | 19 (0.7) | 0 (0.0) |
| Acute mono blastic/cytic leukemia | 6 (0.2) | 2 (0.2) |
| Other monocytic leukemia | 12 (0.4) | 4 (0.3) |
| Other leukemia | 17 (0.6) | 0 (0.0) |
| Histiocytosis | 0 (0.0) | 1 (0.1) |
| Multiple myeloma | 93 (3.3) | 11 (0.9) |
| Myelodysplastic syndrome | 57 (2.0) | 11 (0.9) |
| Myelofibrosis | 7 (0.3) | 1 (0.1) |
| Essential thrombocythemia | 3 (0.1) | 1 (0.1) |
| Other malignant immunoproliferative diseases | 23 (0.8) | 4 (0.3) |
| Kaposi’s sarcoma | 2 (0.1) | 41 (3.3) |
| Autologous stem cell transplantation | 29 (1.0) | 8 (0.6) |
| Allogenic stem cell transplantation | 124 (4.4) | 28 (2.2) |
| **Solid organ cancers** | **606 (21.7)** | **65 (5.2)** |
| Lung cancer | 239 (8.6) | 21 (1.7) |
| Breast cancer | 106 (3.8) | 9 (0.7) |
| Eso gastric cancer | 51 (1.8) | 5 (0.4) |
| Prostatis cancer | 50 (1.8) | 4 (0.3) |
| Anorectal, colon cancer | 22 (0.8) | 3 (0.2) |
| Liver gallbladder, biliary duct, pancreas cancer | 47 (1.7) | 7 (0.6) |
| Brain cancer | 43 (1.5) | 4 (0.3) |
| Kidney cancer | 14 (0.5) | 2 (0.2) |
| Skin melanoma | 14 (0.5) | 2 (0.2) |
| Bladder cancer | 8 (0.3) | 2 (0.2) |
| Larynx cancer | 8 (0.3) | 2 (0.2) |
| Oropharynx cancer | 7 (0.3) | 1 (0.1) |
| Small bowel cancer | 6 (0.2) | 0 (0.0) |
| Ovary cancer | 7 (0.3) | 0 (0.0) |
| Corpus uteri cancer | 3 (0.1) | 2 (0.2) |
| Cervix uteri cancer | 4 (0.1) | 0 (0.0) |
| Bone marrow failure, including profound neutropenia **^a^** | 518 (18.5) | 183 (14.5) |

**Table S2 (*continued*)**

|  | **HIV-negative patients**  N=2794 | **HIV-positive patients**  N=1261 |
| --- | --- | --- |
| **Inflammatory and autoimmune diseases** | **555 (19.9)** | **134 (10.6)** |
| Rheumatoid arthritis | 181 (6.5) | 16 (1.3) |
| Wegener granulomatosis and other necrotizing angiitis | 84 (3.0) | 17 (1.3) |
| Nephrotic, nephritic syndrome | 56 (2.0) | 23 (1.8) |
| Crohn disease | 30 (1.1) | 8 (0.6) |
| Polymyositis, dermatomyositis | 31 (1.1) | 6 (0.5) |
| Sarcoidosis | 29 (1.0) | 4 (0.3) |
| Ulcerative colitis | 20 (0.7) | 11 (0.9) |
| Systemic lupus erythematosus | 18 (0.6) | 6 (0.5) |
| Autoimmune hepatitis | 16 (0.6) | 8 (0.6) |
| Systemic sclerosis | 18 (0.6) | 6 (0.5) |
| Myasthenia gravis | 19 (0.7) | 3 (0.2) |
| Idiopathic thrombocytopenic purpura | 19 (0.7) | 3 (0.2) |
| Horton | 18 (0.6) | 2 (0.2) |
| Polymyalgia rheumatica | 18 (0.6) | 1 (0.1) |
| Cryoglobulinemia | 10 (0.4) | 9 (0.7) |
| Sjögren syndrome | 14 (0.5) | 4 (0.3) |
| Psoriasis | 11 (0.4) | 7 (0.6) |
| Ankylosing spondylarthritis | 13 (0.5) | 2 (0.2) |
| Mixed connective tissue disease | 12 (0.4) | 0 (0.0) |
| Psoriatic arthritis | 5 (0.2) | 1 (0.1) |
| Still disease | 5 (0.2) | 0 (0.0) |
| Pemphigus vulgaris | 3 (0.1) | 0 (0.0) |
| Behcet disease | 1 (0.0) | 0 (0.0) |
| **Solid organ transplantation** | **345 (12.3)** | **119 (9.4)** |
| Kidney transplantation | 220 (7.9) | 78 (6.2) |
| Liver transplantation | 70 (2.5) | 24 (1.9) |
| Heart transplantation | 36 (1.3) | 8 (0.6) |
| Lung transplantation | 18 (0.6) | 9 (0.7) |
| Other transplantation | 36 (1.3) | 6 (0.5) |
| **Idiopathic lung fibrosis** | **121 (4.3)** | **29 (2.3)** |
| **Miscellaneous other causes of immunosuppression ^b^** | **79 (2.8)** | _ |
| **Lack of identified cause of immunodeficiency** | **286 (10.2)** | _ |

**^a^**: Number of patients with at least one type of hematologic malignancy. It should be noted that in the following list, the sum of each category exceeds the number of patients with at least one type of hematologic malignancy. This discrepancy arises from instances where the same malignancy may have been coded differently multiple times during a hospital stay, or because patients may actually have had multiple types of hematologic malignancies. A similar phenomenon occurs with solid organ cancers (as some patients may have had multiple cancers), organ transplantation (where some patients received multiple organ transplants), and inflammatory and autoimmune diseases (as some patients may have had multiple conditions within this category).

**^b^**: Concerns patients with hematologic malignancy or solid organ cancer

**^c^**: Including immunodeficiency mentioned but not specified (n=21)

**Figure S2**

**Figure S2 caption:**

**Title:** Cross-validation of the multivariable, binary logistic model

**Figure S3**

**Figure S3 caption:**

**Title:** Interaction between hematologic malignancy and HIV infection

**Figure S4**

**Figure S4 caption:**

**Title:** Interaction between age and congestive heart failure

**Figure S5**

**Figure S5 caption:**

**Title:** Interaction between age and coagulopathy

**Figure S6**

**Figure S6 caption:**

**Title:** Interaction between age and solid organ cancer

**References**

1. Dunkler D, Plischke M, Leffondré K, Heinze G. Augmented backward elimination: a pragmatic and purposeful way to develop statistical models. PLoS One. 2014 Nov 21;9(11):e113677. doi: 10.1371 journal.pone.0113677.
2. Heinze G, Wallisch C, Dunkler D. Variable selection - A review and recommendations for the practicing statistician. Biom J. 2018 May;60(3):431-449. doi: 10.1002/bimj.201700067.
